# The Association between Circadian Clock Gene Polymorphisms and Metabolic Syndrome: A Systematic Review and Meta-Analysis

**DOI:** 10.3390/biology11010020

**Published:** 2021-12-24

**Authors:** Ivana Škrlec, Jasminka Talapko, Snježana Džijan, Vera Cesar, Nikolina Lazić, Hrvoje Lepeduš

**Affiliations:** 1 Faculty of Dental Medicine and Health Osijek, Josip Juraj Strossmayer University of Osijek, 31000 Osijek, Croatia; jtalapko@fdmz.hr (J.T.); sdzijan@gmail.com (S.D.); vera.cesar@biologija.unios.hr (V.C.); nlazic@fdmz.hr (N.L.); hlepedus@yahoo.com (H.L.); 2 Genos Ltd., DNA Laboratory, 10000 Zagreb, Croatia; 3 Department of Biology, Josip Juraj Strossmayer University of Osijek, Ul. Cara Hadrijana 8/A, 31000 Osijek, Croatia; 4 Faculty of Humanities and Social Sciences Osijek, Josip Juraj Strossmayer University of Osijek, 31000 Osijek, Croatia

**Keywords:** circadian clock genes, hypertension, metabolic syndrome, type 2 diabetes mellitus, obesity

## Abstract

**Simple Summary:**

Metabolic syndrome is a cluster of cardio-metabolic risk factors and comorbidities, including central obesity, hypertension, hyperglycemia, and dyslipidemia. In addition, different studies have shown that the disturbances in circadian rhythm are connected with components of metabolic syndrome. Circadian rhythm is the central regulator of every aspect of human health and metabolism, and metabolic homeostasis is essential in regulating energy metabolism, especially in adipose tissue. Therefore, we aimed to evaluate the association of genetic variations of circadian rhythm genes with metabolic syndrome and its components by a systematic review and meta-analysis. Our findings suggest that some variants of the circadian rhythm gene might be genetic biomarkers applied to predict metabolic syndrome susceptibility.

**Abstract:**

Metabolic syndrome (MetS) is a combination of cardiovascular risk factors associated with type 2 diabetes, obesity, and cardiovascular diseases. The circadian clock gene polymorphisms are very likely to participate in metabolic syndrome genesis and development. However, research findings of the association between circadian rhythm gene polymorphisms and MetS and its comorbidities are not consistent. In this study, a review of the association of circadian clock gene polymorphisms with overall MetS risk was performed. In addition, a meta-analysis was performed to clarify the association between circadian clock gene polymorphisms and MetS susceptibility based on available data. The PubMed and Scopus databases were searched for studies reporting the association between circadian rhythm gene polymorphisms (ARNTL, BMAL1, CLOCK, CRY, PER, NPAS2, REV-ERBα, REV-ERBβ, and RORα) and MetS, and its comorbidities diabetes, obesity, and hypertension. Thirteen independent studies were analyzed with 17,381 subjects in total. The results revealed that the BMAL1 rs7950226 polymorphism was associated with an increased risk of MetS in the overall population. In contrast, the CLOCK rs1801260 and rs6850524 polymorphisms were not associated with MetS. This study suggests that some circadian rhythm gene polymorphisms might be associated with MetS in different populations and potentially used as predictive biomarkers for MetS.

## 1. Introduction

Metabolic syndrome (MetS) is a set of cardiometabolic disorders associated with cardiovascular risk factors [1,2]. Metabolic syndrome is a combination of at least three metabolic disorders that include elevated blood triglyceride levels, decreased HDL levels (high-density lipoprotein cholesterol), elevated fasting glucose levels, and high blood pressure [3]. MetS is thought to underlie obesity and insulin resistance, with central obesity involved in developing type 2 diabetes and cardiovascular diseases [2,4]. Due to a sedentary lifestyle, decreased physical activity and increased obesity remarkably contribute to type 2 diabetes development [2,3]. Moreover, sex, age, and lifestyle habits, such as drinking, tobacco smoking, and educational level could affect metabolic health regardless of obesity [5,6]. MetS negatively affects many body systems. Thus, insulin resistance causes microvascular damage leading to endothelial dysfunction and hypertension. Endothelial damage can cause atherosclerosis and hypertension, negatively affecting peripheral circulation and the heart and leading to kidney damage. In addition, MetS-associated dyslipidemia can trigger atherosclerosis that could lead to ischemic heart disease [3]. 

Circadian rhythm is a significant regulator of every aspect of human health and metabolism, and metabolic homeostasis is vital in regulating energy metabolism, especially in adipose tissue [4,7]. The central circadian clock is established in the hypothalamus’s suprachiasmatic nucleus (SCN) and determines diurnal rhythms [4,8]. The primary activators of transcription are CLOCK and BMAL1 proteins that heterodimerize each other, bind to enhancers, and rhythmically induce the transcription of other circadian clock genes. Period (*PER*) and cryptochrome (*CRY*) genes make up the negative feedback loop. Additionally, BMAL1 stimulates the expression of RORα and γ genes (RAR-orphan receptor). Casein kinases I δ and ε (CKI δ and ε), which degrade CRY and PER proteins, also contribute to the circadian activation of the clock gene. In addition to CRY and PER proteins, REV-ERB proteins (reverse erythroblastosis virus) prevent *BMAL1* expression. Molecular clocks exist in all body cells and show 24 h periodicity [9,10,11]. 

A circadian metabolic disorder is a new risk factor for MetS. Many animal studies have investigated the relationship between circadian rhythm and metabolism. Circadian rhythm disorder has been associated with obesity, diabetes, hypertension, cardiovascular disease, and all components of MetS [4,12,13]. In addition, circadian rhythm disorders can lead to metabolic disorders, such as dyslipidemia, obesity, hyperglycemia, and hypertension [9,14,15]. 

Lipids and glucose have an essential role in developing MetS because they are an integral part of metabolic pathways. Circadian rhythm has a vital function in the homeostasis of lipid and glucose linked to obesity. Thus, it has a role in the risk of developing insulin sensitivity, diabetes, and cardiovascular diseases [4,16]. In addition, it has been shown that some of the core circadian rhythm genes are involved in maintaining lipid and glucose homeostasis, and circadian rhythm disorders may contribute to the development of metabolic health problems [10,17]. 

Many studies that have shown an association between circadian rhythm genes and metabolic syndrome have been conducted in animal models, most commonly in mice and rats that are nocturnal animals. The results of such research are applied to diurnal humans. However, there have been some inconsistencies among studies performed to date. Thus, Cho et al. showed that REV-ERBα alpha was associated with hyperglycemia and high triglyceride levels [18]. In contrast, Solt et al. showed that REV-ERBα alpha prevents weight gain by reducing fat mass [19]. 

Furthermore, the modern lifestyle contributes to reduced light exposure during the day (low indoor lighting levels) and increased light exposure at night, directly affecting our circadian rhythm and health, including the onset of metabolic syndrome. Therefore, the present meta-analysis was conducted to elucidate the association between circadian clock gene polymorphisms and MetS susceptibility. Furthermore, the study might provide novel potential biomarkers for the prognosis of MetS risk. 

## 2. Methods

### 2.1. Literature Search

The databases (Scopus and PubMed) were searched for studies investigating the association between circadian clock gene polymorphisms and metabolic syndrome and its risk factors, such as diabetes, hypertension, and obesity until 6 July 2021. The searching strategy is presented in Supplementary Table S1. The databases search was conducted following PRISMA guidelines (Supplementary File S1), and the study protocol is registered in PROSPERO (CRD42021291934).

### 2.2. Eligibility Criteria

All retrieved articles were primarily selected for acceptable studies regarding specific criteria: cross-sectional or case-control studies and focused on the association of circadian clock gene polymorphisms with MetS risk and its comorbidities. The exclusion criteria included duplicate records, no control group, and departure from Hardy–Weinberg equilibrium in the control group. In addition, only articles written in English were considered. 

### 2.3. Data Extraction 

Two investigators (I.Š., N.L.) independently extracted data. Data on the author, publication year, study design, demographic characteristics, risk factors related to MetS, genotyping method, allele frequencies, distribution of genotypes, diagnostic criteria, and clinical characteristics were collected. Discrepancies were resolved by discussion. The quality of the studies evaluated by the Newcastle-Ottawa Scale (NOS) is based on the range from one to nine stars and three components: selection, comparability, and outcome ascertainment [20]. Studies with a higher number of stars were supposed to be of higher quality.

### 2.4. Statistical Analyses

The principal outcome was the odds ratio (OR) and 95% confidence interval (95% CI) of specific polymorphism in patients with risk factors related to MetS compared to the controls under allelic, dominant, and recessive genetic models. The selection bias was estimated by computing the Hardy–Weinberg equilibrium (HWE) and frequencies of the genotypes. A random-effect model was used because the studies were performed in a wide range of settings in different populations. The considerable between-study heterogeneity is anticipated in genetic association studies. The heterogeneity was estimated using the Cochran Q-test and the I-squared statistic, and I^2^ higher than 50% was considered significant heterogeneity. The sensitivity analysis was conducted to evaluate studies with high statistical heterogeneity by sequentially removing each study to detect pooled results’ stability and heterogeneity source. A meta-analysis was conducted if at least two independent studies for the same polymorphism were available. In addition, publication bias was assessed by Begg’s and Egger’s tests and visual assessment of the funnel plot. A two-sided *p*-value < 0.05 was deemed significant. The Comprehensive Meta-analysis software version 3.3.070 (Biostat Inc., Englewood, NJ, USA) was used for statistical data processing. 

## 3. Results

### 3.1. Characteristics of Eligible Studies

**Figure 1:**
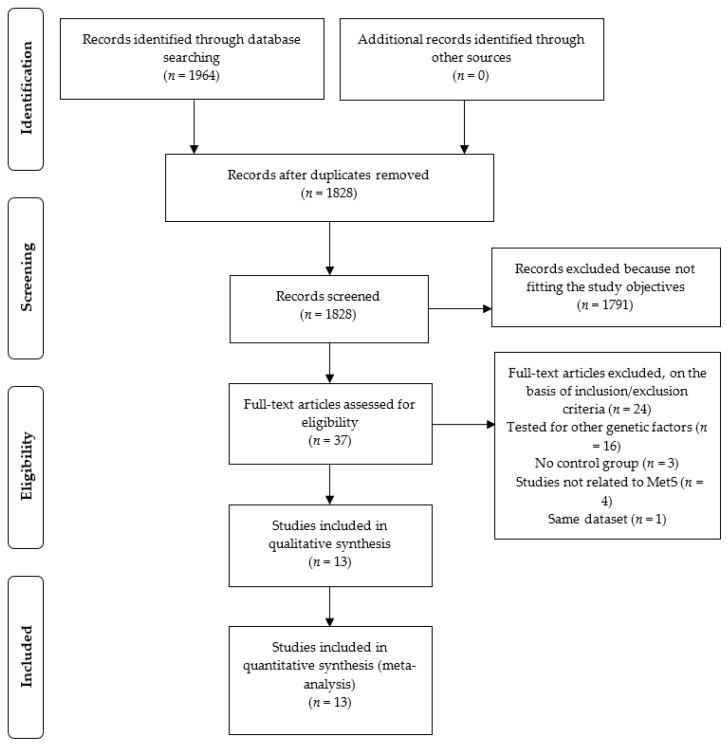
Flowchart of the systematic review on metabolic syndrome.

A sum of 1828 articles was recovered through databases search after removing duplicate articles. Screening titles and abstracts excluded the 1791 articles. Furthermore, 24 articles were excluded based on the inclusion/exclusion criteria. Finally, 13 studies [21,22,23,24,25,26,27,28,29,30,31,32,33] were included in the meta-analysis, analyzing 17,381 subjects in total, including 8726 cases and 8655 controls. The study selection process is presented in Figure 1. Quality scores of the chosen studies ranged from six to eight (Supplementary Table S2). In addition, the characteristics of suitable studies are shown Table 1, while clinical characteristics are in Supplementary Table S3. Among the eligible studies, seven were case-control studies, and six were cross-sectional.

### 3.2. Characteristics of the Circadian Rhythm Gene Polymorphism

In 13 studies included, 11 circadian rhythm genes were analyzed with 46 different polymorphisms. Six studies evaluated the *CLOCK* gene polymorphisms in 3644 participants with MetS risk factors and 3884 control participants [21,22,25,27,29]. Three studies evaluated the *BMAL1* polymorphisms in 3644 participants with MetS risk factors and 3884 control participants [25,27,31]. Three studies evaluated the *PER3* gene polymorphisms in 2156 participants with MetS risk factors and 2247 control participants [25,26,33]. Two studies evaluated the *CRY1* [25,29], *CRY2* [23,25], and *REV*-*ERBα* [28,32] gene polymorphisms. The genotype and allele frequencies of all polymorphisms are presented in Table 2. The majority of the articles and polymorphisms were excluded from the quantitative synthesis for several reasons. Nine polymorphisms in three studies showed selection bias in the control group when assuming Hardy–Weinberg equilibrium [27,28,32]. Many studied polymorphisms were analyzed only in one study [23,26,28,30,32,33]. Finally, only three polymorphisms, *BMAL1* rs7950226, *CLOCK* rs1801260, and rs6850524, were incorporated in the final meta-analysis.

### 3.3. Quantitative Data Synthesis

The association of *BMAL1* and *CLOCK* polymorphisms with overall MetS was estimated by computing pooled ORs. *CLOCK* polymorphisms, rs1801260, and rs6850524, were not associated with MetS risk (*p* = 0.164, OR 1.58, 95% CI 0.83–3.01, and *p* = 0.989, OR 1.00, 95% CI 0.56–1.77, respectively). In contrast, *BMAL1* rs7950226 was linked with overall MetS risk (*p* = 0.007, OR 1.28, 95% CI 1.07–1.54) (Supplementary Figure S1).

Further genetics model analyses were conducted. The *BMAL1* rs7950226 polymorphism could lower the risk for MetS comorbidities (G vs. A *p* = 0.047, OR 0.79, 95% CI 0.62–1.00; GG vs. GA + AA *p* = 0.037, OR 0.75, 95% CI 0.58–0.98). In contrast, *CLOCK* rs6850524 polymorphism did not influence the risk for MetS (Table 3).

Four studies evaluated the rs180126 *CLOCK* gene polymorphisms in 1261 participants with MetS risk factors and 1129 control participants [21,24,27,31], while only two studies evaluated rs6850524 in *CLOCK* [22,29] and rs7950226 in *BMAL1* gene [25,31] with 651 and 1835 participants with MetS risk factors and 975 and 2011 control participants, respectively. The stratified analysis was conducted based on ethnicity and MetS risk factors only for CLOCK rs1801260 polymorphism due to the small number of articles for BMAL1 rs7950226 and CLOCK rs6850524 polymorphisms. Genetics model analyses of the *CLOCK* rs1801260 polymorphism are presented in Figure S2. Polymorphism rs1801260 in the *CLOCK* gene showed significant association in some particular subgroups (Supplementary Table S4). Although only one study was done in the Asian population, the rs1801260 variant could increase metabolic syndrome risk in the mentioned population (Supplementary Table S4). Likewise, as a component of MetS, hypertension is less likely to lead to MetS. In contrast, insulin resistance is strongly associated with MetS (Supplementary Table S4).

### 3.4. Sensitivity Analysis

Sensitivity analysis was performed to evaluate the weight of some single study on pooled effects by determining the ORs before and after removing individual research from a meta-analysis. After being excluded, no outlying article was recognized to modify the pooled ORs substantially (Supplementary Table S5).

### 3.5. Publication Bias

Moreover, the possible publication bias was estimated for all included studies utilizing Begg’s and Egger’s tests. No significant publication bias was confirmed in any genetics model of *CLOCK* rs1801260 polymorphism (Table 4). In addition, the funnel plots of log odds ratio versus standard error were proportional (Supplementary Figure S3).

## 4. Discussion

In the present study, a systematic review was conducted to associate circadian clock gene polymorphisms with the overall risk of metabolic syndrome. In addition, a meta-analysis was conducted for three frequently investigated polymorphisms in *CLOCK* (rs1801260 T > C and rs6850524 G > C) and *BMAL1* gene (rs7950226 G > A). The results revealed that *BMAL1* rs7950226 polymorphism was linked with MetS risk in the overall population, while *CLOCK* rs1801260 polymorphism was associated with some specific subgroups. Thus, it is the first time comprehensively assessing the study progress in this domain and the first meta-analysis of MetS-related circadian clock polymorphisms.

CLOCK protein is included in the transcriptional control of circadian output genes and the core circadian clock. Hence, up to 10% of the human transcriptome may be under circadian regulation, and disorder in the CLOCK gene substantially influences transcription control.

The *CLOCK* rs1801260 (T3111C) polymorphism, placed in the 3′-UTR of the gene, has been extensively studied for its function in different MetS risk factors and in MetS patients on various diets. It is the first polymorphism recognized in the *CLOCK* gene to be linked with human MetS phenotypes [34]. However, although some studies observed a notable association between rs1801260 polymorphism and MetS susceptibility, the present meta-analysis did not find an association to overall MetS risk. That might happen due to the insufficient number of participants, the ethnic diversity of the studied population, and complex environmental circumstances that vary depending on the study [35]. However, insulin resistance is strongly associated with most risk factors related to MetS in all tested genetic models (allelic, dominant, and recessive). In contrast, hypertension as a sole risk factor is less likely to lead to MetS (Table S4). Moreover, the importance of the rs1801260 polymorphism with MetS risk was observed in the Asian subgroup rather than in the Caucasian and Hispanic subgroups in stratified analysis. The C allele was an independent risk factor for potential insulin resistance in Asian patients with essential hypertension [31] and diabetes [36], while in Caucasian patients was positively associated with hypertension and coronary artery disease and negatively with obesity [27,37,38]. Monteleone et al. [12] noted that the rs1801260 genotypes were not linked with obesity. However, they observed a significant connection of the rs1801260 genotypes among overweight participants. Li et al. [15] observed that patients with the C allele incline towards insulin resistance and MetS, which could lead to the discrepancy. The rs1801260 polymorphism within 3′-UTR could be accountable for changes in the secondary structure of mRNA. Thus, polymorphism within 3′-UTR polymorphism results in various mRNA’s secondary structures that interfere with RNA-binding proteins and miRNA-182 binding sites in the 3′-UTR [39]. The *CLOCK* rs1801260 polymorphism might influence its transcription by modifying mRNA durability and then participating in MetS development. Ozburn et al. showed that rs1801260 polymorphism alters the expression, function, and stability of *CLOCK* mRNA and consequently affects the *PER2* expression [40]. In conclusion, the abovementioned findings showed that *BMAL1* rs7950226 polymorphism might be a predictive biomarker for MetS risk in the overall population. Nevertheless, additional molecular analyses require elucidation of all the assumptions regarding relevant mechanisms.

Unlike *CLOCK* rs1801260, rs6850524 polymorphism is an intron variant with three possible variations: C > G, C > A, and C > T, with C > G being the most common variant. Although studies revealed a discrepancy in the association of *CLOCK* rs6850524 polymorphism and MetS risk factors, this meta-analysis observed no association. However, some studies found an increased risk for hypertension [37] and obesity [22,29] associated with rs6850524. Still, additional studies are needed to investigate the molecular mechanism involved.

Similar to *CLOCK* rs6850524 polymorphism, *BMAL1* rs7950226 polymorphism is an intron variant G > A and has been extensively studied for its function in different MetS risk factors. In the present study, a statistically significant association of *BMAL1* rs7950226 with MetS risk was observed in the overall population (Table 3). Based on the results of this meta-analysis, it has been revealed that carriers of the A allele or AA genotype have an elevated risk of developing MetS. Furthermore, Pappa et al. found that the A allele is significantly linked with a higher risk of gestational diabetes mellitus [41], while other studies showed an association with type 2 diabetes mellitus [25,42]. Moreover, rs7950226 GG genotype was associated with insulin resistance in patients with hypertension [31]. First, though, the molecular mechanisms underlying MetS need to be further clarified.

As observed in this study, *CLOCK* and *BMAL1* gene polymorphisms are associated with some components of MetS. Polymorphisms within other circadian rhythm genes are also linked with elements of MetS. *CRY1* and *CRY2* polymorphisms showed a significant link between diabetes and obesity [23,25,29,43]. *CRY1* polymorphisms are negatively associated with obesity [29]. Recent studies reported that *PER2* and *PER3* polymorphisms are associated with diabetes and obesity, metabolic syndrome components [25,26,33]. *PER2* polymorphisms are negatively associated with diabetes, while *PER3* polymorphisms are positively associated with obesity. Moreover, some studies observed a link between circadian gene *REV-ERBα*, *REV-ERBβ*, and *RORα* polymorphisms and obesity and diabetes [28,30,32]. *RORα* variants are linked with an increased possibility of developing diabetes [30], while *PER3* and *RORα* polymorphisms increase the risk of MetS in the Taiwanese population [16]. However, those articles were not incorporated in the quantitative investigation due to a limited number of the same polymorphisms investigated. In addition, circadian rhythm genes might contribute to the risk of MetS independently and via gene-gene and gene-environment interplays. Additionally, to core clock genes, other genes expressed in a circadian manner and lifestyle could affect susceptibility to metabolic syndrome. For example, melatonin is a hormone responsible for regulating circadian rhythm and might affect glucose metabolism, associated with insulin resistance and T2DM and, therefore, MetS [44,45]. Moreover, genetic variants of melatonin receptor 1B (*MTNR1B*) might affect melatonin function and influence susceptibility to insulin resistance and diet-dependent weight loss [46,47]. Some studies suggest that genetic variants of core clock genes under a specific diet could influence risk factors for MetS, such as glucose levels, dyslipidemia, T2DM [48,49]. However, further similar research is required to be included in the meta-analysis to elucidate the precise link between the different circadian clock gene polymorphisms and the overall MetS risk.

The present study has some shortcomings. First, the database search was limited to English articles, so that some studies could have been overlooked. In addition, although the meta-analysis included a moderate number of participants, the critical studies are somewhat limited. Thus, this area requires further research at the molecular level and updated meta-analysis.

## 5. Conclusions

In conclusion, the association of circadian clock gene polymorphisms with the overall risk of MetS was reviewed. Moreover, a meta-analysis was conducted employing all possible data for three often studied polymorphisms among them (*CLOCK* rs1801260 and rs6850524, and *BMAL1* rs7950226). The results revealed that the *BMAL1* rs7950226 polymorphism was linked with the risk of MetS in the overall population. In contrast, the *CLOCK* rs1801260 polymorphism was associated with particular subgroups, implying it might be a possible prognostic biomarker for MetS risk. Thus, the study might present new hints to identify genetic biomarkers related to miRNAs that predict MetS susceptibility.

## Figures and Tables

**Table 1:** Characteristics of studies included in the meta-analysis.

First Author	Year	Country	Ethnicity	Study Type	Risk Factor	Population Type	Age Cases	Age Controls	Case	Control	Male (%)	Genotyping Method	Tested Genes	SNPs
Monteleone et al. [21]	2008	Italy	Caucasian	Case-control	Obesity	general	38.4 ± 10.9	26.1 ± 4.6	192	92	14.79%	RFLP-PCR	CLOCK	rs1801260
Sookoian et al. [22]	2008	Brazil	Hispanic	Cross-sectional	Obesity	general	37.55 ± 0.45	32.66 ± 0.29	391	715	0.00%	PCR	CLOCK	rs1554483 rs11932595 rs4580704 rs6843722 rs6850524 rs4864548
Hu et al. [23]	2010	China	Asian	Case-control	T2DM	general	60.33 ± 12.94	50.10 ± 14.27	3410	3412	47.42%	MassArray	CRY2	rs11605924
Galbete et al. [24]	2012	Spain	Hispanic	Cross-sectional	Obesity	general	70 ± 6	67 ± 5	532	371	72.76%	real-time PCR	CLOCK	rs1801260
Kelly et al. [25]	2012	UK/Pakistan	Asian	Case-control	T2DM	general	55.94 ± 11.88	55.8 ± 11.28	1732	1780	49.32%	real-time PCR	BMAL1 CLOCK CRY1 CRY2 NPAS2 PER1 PER2 PER3	rs11022775 rs7950226 rs11133373 rs12315175 rs2292912 rs1369481 rs17024926 rs895521 rs2289591 rs885747 rs7602358 rs1012477
Karthikeyan et al. [26]	2014	India	Asian	Case-control	T2DM	general	50.7 ± 10.3	49.9 ± 9.1	302	330	58.23%	PCR	PER3	4/5–VNTR
Kolomeichuk et al. [27]	2014	Russia	Caucasian	Cross-sectional	Hypertension	general	51.9 ± 6.9	50.8 ± 8.1	434	435	48.33%	RFLP-PCR	BMAL1 CLOCK	rs6486121 rs1801260 rs4865010 rs34789226 rs3736544
Ruano et al. [28]	2014	Spain	Hispanic	Cross-sectional	Obesity	general	64.33 ± 9.0	62.7 ± 8.9	779	418	40.10%	real-time PCR	REV-ERBα	rs939347 rs2071427
Ye et al. [29]	2016	China	Asian	Cross-sectional	Obesity	general	52.09 ± 8.22	52.09 ± 8.21	260	260	48.85%	MassArray	CLOCK CRY1	rs10002541 rs6850524 rs10861688
Zhang et al. [30]	2016	China	Asian	Case-control	T2DM	hospital	57.37 ± 11.28	58.26 ± 10.51	427	408	51.26%	SNaPshot	RORα	rs17270188 rs1898413 rs11638541 rs8033552 rs10851685 rs8041381 rs340002 rs340023 rs28724570
Li et al. [31]	2020	China	Asian	Cross-sectional	Insuline resistance	general	54 ± 13.81	53.10 ± 11.27	103	231	57.80%	sqeuncing	CLOCK BMAL1	rs1801260 rs7950226
Tokat et al. [32]	2020	Turkey	Caucasian	Case-control	T2DM	general	59.2 ± 1.3	59.0 ± 3.0	42	66	42.59%	NGS	REV-ERBα REV-ERBβ	chr17:38253751T > C rs72836608 rs2314339 rs2102928 chr3:24003765A > G rs924403442
Guimarães de Azevedo et al. [33]	2021	Brazil	Caucasian	Case-control	Obesity	hospital	42.69 ± 15.85	54.5 ± 21.2	122	137	25.10%	real-time PCR	PER3	rs707467 rs228697 rs228729

**Table 2:** Allele and genotype frequency on the circadian rhythm SNPs.

First Author	Tested Genes	SNPs	MAF Allele	MAF Cases	MAF CTRL	Wild Homozygote CTRL	Heterozygote CTRL	Variant Homozygote CTRL	Wild Homozygote Cases	Heterozygote Cases	Variant Homozygote Cases	HWE *p*-Value	Included in Meta-Analysis
Monteleone 2008	CLOCK	rs1801260 T > C	C	0.291	0.288	46	39	7	103	68	21	0.75	Yes
Sookoian 2008	CLOCK	rs1554483 C > G	G	0.465	0.408	251	337	123	111	192	86	0.58	No ^a^
		rs11932595 A > G	G	0.651	0.637	93	323	294	48	173	168	0.77	No ^a^
		rs4580704 C > G	G	0.711	0.678	72	303	333	35	146	205	0.8	No ^a^
		rs6843722 A > C	C	0.432	0.382	273	322	112	123	192	73	0.29	No ^a^
		rs6850524 G > C	C	0.662	0.606	100	306	280	47	146	186	0.27	Yes
		rs4864548 G > A	A	0.457	0.404	248	336	121	111	191	83	0.69	No ^a^
Hu 2010	CRY2	rs11605924 C > A	A	0.245	0.230	181	1210	2021	205	1261	1944	0.99	No ^a^
Galbete 2012	CLOCK	rs1801260 T > C	C	0.305	0.273	181	154	36	278	217	37	0.69	Yes
Kelly 2012	BMAL1	rs11022775 C > T	T	0.19	0.16	1256	478	46	1136	533	63	0.95	No ^a^
		rs7950226 G > A	A	0.5	0.46	519	884	377	433	866	433	0.99	Yes
	CLOCK	rs11133373 C > G	G	0.4	0.38	684	839	257	624	831	277	0.99	No ^a^
	CRY1	rs12315175 T > C	C	0.06	0.07	1540	232	9	1530	195	6	0.93	No ^a^
	CRY2	rs2292912 G > C	C	0.25	0.27	949	702	130	974	650	108	0.99	No ^a^
	NPAS2	rs1369481 C > T	T	0.23	0.24	1028	649	103	1027	613	92	0.97	No ^a^
		rs17024926 T > C	C	0.29	0.32	823	775	182	873	713	146	0.98	No ^a^
		rs895521 C > T	T	0.14	0.15	1286	454	40	1281	417	34	0.99	No ^a^
	PER1	rs2289591 C > A	A	0.17	0.14	1316	429	35	1193	489	50	0.99	No ^a^
		rs885747 G > C	C	0.23	0.29	897	733	150	1027	613	92	0.99	No ^a^
	PER2	rs7602358 T > G	G	0.15	0.16	1256	478	46	1251	442	39	0.95	No ^a^
	PER3	rs1012477 G > C	C	0.05	0.05	1606	169	4	1563	165	4	0.84	No ^a^
Karthikeyan 2014	PER3	VNTR–4/5	5-	0.43	0.35	136	155	39	102	143	57	0.61	No ^a^
Kolomeichuk 2014	BMAL1	rs6486121 T > C	C	0.479	0.451	135	204	96	117	217	100	0.054	No ^a^
	CLOCK	rs1801260 T > C	C	0.419	0.310	209	187	39	143	213	78	0.76	Yes
		rs4865010 T > G	T	0.472	0.629	187	170	78	139	139	156	*<0.001*	No ^a,b^
		rs34789226 T > C	T	0.41	0.531	109	244	83	87	178	169	*0.01*	No ^a,b^
		rs3736544 G > A	G	0.449	0.409	109	139	187	126	135	174	*<0.001*	No ^a,b^
Ruano 2014	REV-ERBα	rs939347 G > A	A	0.207	0.199	261	146	10	494	241	41	*0.045*	No ^a,b^
		rs2071427 C > T	T	0.237	0.25	235	157	26	453	274	48	0.97	No ^a^
Ye 2016	CLOCK	rs10002541 T > C	C	0.271	0.335	117	112	31	134	104	17	0.59	No ^a^
		rs6850524 G > C	C	0.268	0.322	117	112	26	134	104	16	0.92	Yes
	CRY1	rs10861688 C > T	T	0.271	0.311	126	102	29	133	106	16	0.23	No ^a^
Zhang 2016	RORα	rs17270188 G > A	A	0.448	0.461	93	190	125	94	195	138	0.2	No ^a^
		rs1898413 G > A	A	0.164	0.156	10	107	291	14	112	301	0.96	No ^a^
		rs11638541 T > C	C	0.115	0.105	327	76	5	336	84	7	0.81	No ^a^
		rs8033552 G > A	A	0.177	0.164	14	106	288	17	117	193	0.28	No ^a^
		rs10851685 A > T	T	0.294	0.191	13	130	265	26	156	245	0.54	No ^a^
		rs8041381 A > G	G	0.144	0.138	299	105	4	310	111	6	0.11	No ^a^
		rs340002 G > A	A	0.358	0.346	43	196	169	51	204	172	0.21	No ^a^
		rs340023 T > C	C	0.381	0.362	56	183	169	68	189	170	0.57	No ^a^
		rs28724570 C > T	T	0.498	0.534	90	200	118	107	215	105	0.76	No ^a^
Li 2020	BMAL1 CLOCK	rs7950226 G > A rs1801260 T > C	A C	0,45 0.108	0,35 0.291	64 186	126 40	41 5	45 51	45 44	14 8	0.12 0.12	Yes Yes
Tokat 2020	REV-ERBα	chr17:38253751T > C	C	0.31	0.288	28	38	0	16	26	0	*0.001*	No ^a,b^
		rs72836608 C > A	A	0.321	0.295	33	6	27	19	4	19	*<0.001*	No ^a,b^
		rs2314339 C > T	T	0.19	0.212	40	2	24	28	2	12	*<0.001*	No ^a,b^
		rs2102928 C > T	T	0.357	0.356	26	7	33	17	5	20	*<0.001*	No ^a,b^
	REV-ERBβ	chr3:24003765 A > G	G	0.143	0.129	47	17	0	30	12	0	0.22	No ^a^
		rs924403442 G > T	T	0.25	0.288	28	38	0	21	21	0	*0.001*	No ^a,b^
Guimarães de Azevedo 2021	PER3	rs707467 A > C	C	0.188	0.236	69	48	5	78	34	5	0.34	No ^a^
		rs228697 C > G	G	0.058	0.041	115	8	1	107	14	0	0.06	No ^a^
		rs228729 C > T	T	0.379	0.293	65	43	14	43	63	14	0.11	No ^a^

MAF—minor allele frequency; CTRL—Controls; HWE—Hardy–Weinberg equilibrium; italic HWE *p* values are statistically significant; a—Excluded due to the insufficient number of the studies; b—excluded due to departure from the HWE in the control group.

**Table 3:** Meta-analysis results of the *BMAL1* rs7950226 and *CLOCK* rs1801260 and rs6850524 polymorphisms and MetS risk.

Comparison	SNP	Test of Association		Test of Heterogeneity		
		OR (95% CI)	*p*	I^2^	Q	*p*
*BMAL1*	rs7950226					
Allelic model	G vs. A	0.79 (0.62–1.00)	0.047	54%	2.18	0.140
Dominant model	GG + GA vs. AA	0.74 (0.54–1.02)	0.680	34%	1.512	0.217
Recessive model	GG vs. GA + AA	0.75 (0.58–0.98)	0.037	34%	1.53	0.216
*CLOCK*	rs1801260					
Allelic model	T vs. C	1.00 (0.61–1.63)	0.506	94%	48.19	<0.001
Dominant model	TT + TC vs. CC	0.99 (0.52–1.89)	0.797	80%	15.03	0.002
Recessive model	TT vs. TC + CC	0.99 (0.52–1.83)	0.548	93%	42.35	<0.001
*CLOCK*	rs6850524					
Allelic model	G vs. C	1.00 (0.61–1.63)	0.96	89%	9.24	0.002
Dominant model	GG + CG vs. CC	0.99 (0.52–1.89)	0.96	74%	3.98	0.046

**Table 4:** Publication bias was assessed by Begg’s and Egger’s tests.

Association	Begg’s Test		Egger’s Test	
	*Z* Value	*p* Value	*t* Value	*p* Value
*CLOCK* rs1801260 T > C	1.69	0.089	2.24	0.154
Allelic model	1.02	0.308	1.33	0.315
Dominant model	1.02	0.308	1.45	0.284
Recessive model	0.339	0.734	1.08	0.393

## Data Availability

The datasets used and/or analyzed during the current study are available from the corresponding author on reasonable request.

## Supplementary Materials

The following are available online at https://www.mdpi.com/article/10.3390/biology11010020/s1, Supplementary File S1: PRISMA 2020 checklist. Table S1: Search strategy: PubMed and Scopus through 6 July 2021. Table S2: Quality assessment of studies included in the meta-analysis by Newcastle-Ottawa scale, Table S3: Clinical characteristics of participants included in the meta-analysis. Table S4: The meta-analysis of the association between *CLOCK* rs1801260 polymorphism and metabolic syndrome risk. Table S5: Sensitivity analysis of the rs1801260 polymorphism in the CLOCK gene. Figure S1: Forest plot for the association between metabolic syndrome and *BMAL1* and *CLOCK* gene polymorphisms. (A) Forest plot for the association between metabolic syndrome and rs7950226 in *BMAL1* gene with the heterogeneity of 28% (Cochran’s Q = 1.39, *p* = 0.24). (B) Forest plot for the association between metabolic syndrome and rs1801260 in *CLOCK* gene with the heterogeneity of 93% (Cochran’s Q = 42.71, *p* < 0.001). (C) Forest plot for the association between metabolic syndrome and rs6850524 in *CLOCK* gene with the heterogeneity of 90% (Cochran’s Q = 9.89, *p* = 0.002). The area of each square is proportional to the weight that the individual study contributed to the meta-analysis. Weights are from the random-effects analysis. Figure S2: Forest plot for the association between metabolic syndrome and *CLOCK* gene rs1801260 polymorphism. Panel (A) Forest plot for the association between metabolic syndrome and allelic model of rs1801260 polymorphisms with the heterogeneity of 85% (Cochran’s Q = 48.18, I^2^ = 93.78%, *p* < 0.001). (B) Forest plot for the association between metabolic syndrome and dominant model of rs1801260 polymorphisms with the heterogeneity of 81% (Cochran’s Q = 15.03, I^2^ = 80.05%, *p* = 0.001). Panel (C) Forest plot for the association between metabolic syndrome and recessive model of rs1801260 polymorphisms with the heterogeneity of 92% (Cochran’s Q = 42.35, I^2^ = 92.92%, *p* < 0.001). The area of each square is proportional to the weight that the individual study contributed to the meta-analysis. Weights are from the random-effects analysis. Figure S3: Funnel plot of meta-analysis of the *CLOCK* gene rs1801260 polymorphism. (A) For the allelic model, (B) for the dominant model, (C) for the recessive model. Black circles denote imputed studies, trim-and-fill adjustment for publication bias.
