# Supplementary material for: The Association between Circadian Clock Gene Polymorphisms and Metabolic Syndrome: A Systematic Review and Meta-Analysis
Source: Biology (Basel). 2021 Dec 24;11(1):20. doi: 10.3390/biology11010020 (PMC8773381; doi:10.3390/biology11010020)
Supplement: Supplementary file 1 [file biology-11-00020-s001.zip › Supplementary Materials (Tables S1-S5 and Figures S1-S3).pdf]

Supplementary Materials

Table S1. Search strategy: PubMed and Scopus through July 6, 2021

Database	Search strategy
**PubMed**	((((((Metabolic syndrome) OR (diabetes)) OR (hypertension)) OR (obesity)) AND ((circadian rhythm) OR (circadian clock))) AND (((single nucleotide polymorphism) OR (SNP)) OR (variant))) AND ((((((((((ARNTL) OR (BAML1)) OR (CLOCK)) OR (cryptochrome)) OR (period)) OR (NPAS2)) OR (RORA)) OR (rev erb alpha)) OR (RORB)) OR (timeless))
**SCOPUS**	(((ALL (*rev* AND *erb* AND *a*)) OR ((ALL (*arntl*)) OR (ALL (*bmal1*)) OR (ALL (*clock*)) OR (ALL (*cryptochrome*)) OR (ALL (*period*)) OR (ALL (*npas2*)) OR (ALL (*rora*)) OR (ALL (*rorb*)) OR (ALL (*timeless*)))) AND ((ALL (*single* AND *nucleotide* AND *polymorphism*)) OR (ALL (*variant*)) OR (ALL (*snp*)))) AND ((A LL (*circadian* AND *rhythm*)) OR (ALL (*circadian* AND *clock*))) AND ((TITLE-ABS-KEY (*metabolic* AND *syndrome*)) OR (TITLE-ABS-KEY (*diabetes*)) OR (TITLE-ABS-KEY (*hypertension*)) OR (TITLE-ABS-KEY (*obesity*)))

Table S2. Quality assessment of studies included in the meta-analysis by Newcastle-Ottawa scale.

First author	Year	Selection				Comparability	Exposure			Overall quality score
		Adequate definition of cases	Representativeness of the cases	Selection of controls	Definition of controls	Comparability of cases and controls based on the design or analysis	Ascertainment of exposure	Same method of ascertainment for cases and controls	Non-response rate	
Monteleone et al. 2008.	2008	*	*	*	*	*	*	*	-	7
Sookoian et al. 2008.	2008	*	*	*	*	*	*	*	-	7
Hu et al. 2010.	2010	*	*	*	*	*	*	*	-	7
Galbete et al. 2012.	2012	*	*	-	*	*	*	*	-	6
Kelly et al. 2012.	2012	*	*	*	*	**	*	*	-	8
Karthikeyan et al. 2014.	2014	*	*	*	*	**	*	*	-	8
Kolomeichuk et al. 2014.	2014	*	*	*	*	**	*	*	-	8
Ruano et al. 2014.	2014	*	*	-	*	**	*	*	-	7
Ye et al. 2016.	2016	*	*	*	*	**	*	*	-	8
Zhang et al. 2016.	2016	*	*	*	*	**	*	*	-	8
Li et al. 2020.	2020	*	*	-	*	*	*	*	-	6
Tokat et al. 2020.	2020	*	*	-	*	**	*	*	-	7
Guimarães de Azevedo et al. 2021.	2021	*	*	-	*	**	*	*	-	7

Table S3. Clinical characteristics of participants included in the meta-analysis.

**First author**	Group	**Monteleone 2008**	**Sookoian 2008**	**Hu 2010**	**Galbete 2012**	**Kelly 2012**	**Karthikeyan 2014**	**Kolomeichuk 2014**	**Ruano 2014**	**Ye 2016**	**Zhang 2016**	**Li 2020**	**Tokat 2020**	**Guimarães de Azevedo 2021.**
Diagnostic criteria		DSM-IV and WHO criteria for BMI	BMI ≥ 27	standard 75g OGTT	BMI ≥ 25	WHO criteria for T2DM	NIDDK criteria for T2DM	SBP>140mmHg and DBP>90 mmHg, or use of antihypertensive drugs	BMI ≥ 27	men with WC ≥ 85 cm and women with WC ≥ 80 cm	WHO criteria for T2DM	HORMA-IR ≥ 2.69	hospital diagnosed T2DM	BMI ≥ 25
Systolic blood pressure (mm Hg)	Cases	ND	126.5±0.8	ND	ND	137±20.7	ND	139.8±9.3	ND	ND	140.2±18.2	133.7±12.9	133.8±9.8	ND
	CTRL	ND	118.9±0.5	ND	ND	131.8±20	ND	108.2±8.0	ND	ND	124.4±14.2	131±10.9	116.9±2.0	ND
Diastolic blood pressure (mm Hg)	Cases	ND	79.4 ± 0. 6	ND	ND	84.1±11.3	ND	92.4±2.9	ND	ND	83.5±8.7	79.2±8.9	81.9±4.2	ND
	CTRL	ND	74.4±0.3	ND	ND	82.8±12.2	ND	77.1±3.2	ND	ND	77.6±9.3	77.1±9.2	75.8±1.4	ND
Total cholesterol (mmol/l)	Cases	ND	5.2 ±0.05	ND	ND	4.8±1.14	ND	6.1±0.11	4.9±1.1	ND	5.1±0.9	5.2±1.3	5.2±0.21	ND
	CTRL	ND	4.9 ±0.03	ND	ND	4.7±1.12	ND	5.3±0.15	5.2±1.4	ND	4.9±0.9	4.9±1.19	5.1±0.2	ND
HDL (mmol/l)	Cases	ND	1.2±0.02	ND	ND	1.2±0.4	ND	1.2±0.04	1.2±0.5	ND	1.2±0.3	1.2±0.3	1.1±0.05	ND
	CTRL	ND	1.2±0.01	ND	ND	1.2±0.3	ND	1.5±0.04	1.5±0.5	ND	1.4±0.47	1.3±0.4	1.3±0.05	ND
LDL (mmol/l)	Cases	ND	3.3±0.05	ND	ND	2.7±1.03	ND	3.9±0.16	2.9±0.9	ND	2.8±0.8	3.4±1.1	3.3±0.17	ND
	CTRL	ND	3.1±0.03	ND	ND	2.9±1.05	ND	3.1±0.13	2.8±0.9	ND	2.7±0.9	3.3±0.9	3.04±0.12	ND
Triglyceride (mmol/l)	Cases	ND	1.7±0.1	ND	ND	2.5±1.8	ND	1.8±0.1	1.7±1.05	ND	1.7±0.5	2.02±1.03	2.07±0.23	ND
	CTRL	ND	1.3±0.03	ND	ND	1.74±1.05	ND	1.4±0.1	1.0±0.5	ND	1.2±0.3	1.5±0.8	1.3±0.09	ND
Fasting glucose (mmol/l)	Cases	ND	5.1±0.04	ND	ND	ND	ND	4.9±0.4	7.8±3.1	ND	9.8±1.2	ND	9.0±0.5	ND
	CTRL	ND	4.9±0.02	5±0.5	ND	5.1±0.7	ND	4.8±0.3	4.9±0.7	ND	4.9±0.6	ND	5.0±0.1	ND
BMI (kg/m^2)	Cases	21.8±3.53	30.9±0.29	24.4±3.5	26.5±3	27.4±4.5	ND	25.1±3.6	34.4±3.1	26.9±2.8	24.2±2.3	25.62±2.77	30.5±0.8	41.9±5.62
	CTRL	40.8±7.63	24.3±0.07	23.5±3.25	24.1±3.45	26.2±4.8	ND	24.3±4.3	23.3±1.5	21.5±1.4	23.3±2.1	23.7±2.7	25.6±0.5	22.9±2.8
Waist circumference (cm)	Cases	ND	103.±0.52	ND	ND	99.6±11.3	ND	ND	111.1±10.1	88.8±5.8	ND	91.3±9.7	105.0±2.1	ND
	CTRL	ND	86.8±0.4	ND	ND	95.9±12.2	ND	ND	82.81±8.1	72.3±5.2	ND	85.6±9.8	82.2±3.4	ND

CTRL – control group; ND – no data; BMI – body mass index; OGTT – oral glucose tolerance test; T2DM – type 2 diabetes mellitus; NIDDK – National Institute of Diabetes and Digestive and Kidney Diseases, Bethesda, MD, USA; SBP – systolic blood pressure; DBP – diastolic blood pressure; WC – waist circumference

Table S4. The meta-analysis of the association between *CLOCK* rs1801260 polymorphism and metabolic syndrome risk.

SNP	N	Cases	CTRL	Allelic model			Dominant model			Recessive model		
				P	OR (95% CI)	I^2 (%)	P	OR (95% CI)	I^2 (%)	P	OR (95% CI)	I^2 (%)
rs1801260 T>C	4	1261	1129	0.506	1.21 (0.69-2.13)	93.8	0.797	1.11 (0.51-2.41)	80.1	0.548	1.23 (0.62-2.43)	92.9
Ethnicity												
Asian	1	103	231	<0.001	3.42 (2.10-5.55)	0	0.028	9.67 (1.28-73.04)	0	<0.001	3.87 (2.25-6.67)	0
Caucasian	2	626	527	0.219	0. 76 (0.49-1.18)	76.3	0.217	0.65 (0.33-1.29)	53.9	0.213	0.71 (0.42-1.21)	72.3
Hispanic	1	532	371	0.138	1.17 (0.95-1.44)	0	0.306	1.28 (0.80-2.06)	0	0.177	1.20 (0.92-1.57)	0
Risk factor												
Hypertension	1	434	435	0.001	0.62 (0.51-0.76)	0	<0.001	0.50 (0.34-0.75)	0	<0.001	0.56 (0.43-0.74)	0
Insulin resistance	1	103	231	<0.001	3.42 (2.10-5.55)	0	0.028	9.67 (1.28-73.04)	0	<0.001	3.87 (2.25-6.67)	0
Obesity	2	724	463	0.206	1.12 (0.94-1.35)	0	0.341	1.23 (0.81-1.86)	0	0.250	1.15 (0.91-1.45)	0

CTRL – subjects in the control group; P was calculated by the random-effect model; OR – odds ratio; CI – confidence interval

Table S5. Sensitivity analysis of the rs1801260 polymorphism in the *CLOCK* gene

	**Allelic model**	**Dominant model**	**Recessive model**
Study	OR (95% CI)	OR (95% CI)	OR (95% CI)
Overall	1.21 (0.69–2.13)	1.11 (0.51–2.41)	1.23 (0.62–2.43)
Monteleone 2008.	1.31 (0.63–2.72)	1.20 (0.43–3.33)	1.34 (0.55–3.24)
Galbete 2012.	1.25 (0.50–3.11)	1.17 (0.36–3.78)	1.26 (0.42–3.77)
Kolomeichuk 2014.	1.53 (0.83–2.82)	1.51 (0.71–3.21)	1.62 (0.79–3.31)
Li 2020.	0.89 (0.57–1.38)	0.85 (0.43–1.68)	0.87 (0.51–1.46)

A) rs7950226

Study name	Odds ratio	Lower limit	Upper limit	p-Value
Kelly 2012.	1,22	1,07	1,39	0,003
Li 2020.	1,52	1,08	2,14	0,016
	1,28	1,07	1,54	0,007

Statistics for each study; Odds ratio and 95% CI

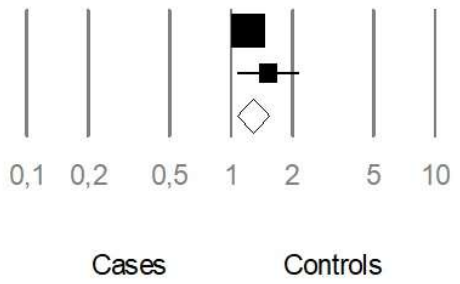

B) rs1801260

Study name	Odds ratio	Lower limit	Upper limit	Z-Value	p-Value
Monteleone 2008.	1,02	0,69	1,50	0,10	0,920
Kolomeichuk 2014.	2,22	1,47	3,35	3,81	0,000
Galbete 2012.	0,86	0,70	1,06	-1,43	0,152
Li 2020.	3,39	2,23	5,16	5,69	0,000
	1,58	0,83	3,01	1,39	0,164

Statistics for each study; Odds ratio and 95% CI

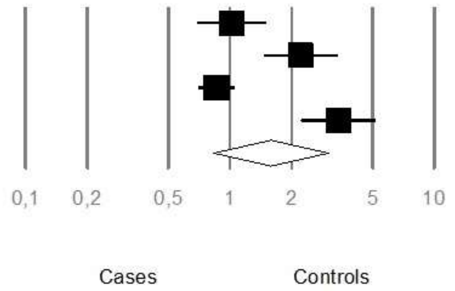

C) rs6850524

Study name	Odds ratio	Lower limit	Upper limit	Z-Value	p-Value
Sookoian 2008.	1,33	1,05	1,69	2,32	0,020
Ye 2016.	0,74	0,56	0,97	-2,15	0,032
	1,00	0,56	1,77	-0,01	0,989

Statistics for each study; Odds ratio and 95% CI

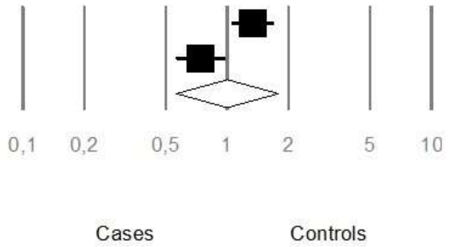

Figure S1. Forest plot for the association between metabolic syndrome and *BMAL1* and *CLOCK* gene polymorphisms. A) Forest plot for the association between metabolic syndrome and rs7950226 in *BMAL1* gene with the heterogeneity of 28% (Cochran's Q=1.39, P=0.24). B) Forest plot for the association between metabolic syndrome and rs1801260 in *CLOCK* gene with the heterogeneity of 93% (Cochran's Q=42.71, P<0.001). C) Forest plot for the association between metabolic syndrome and rs6850524 in *CLOCK* gene with the heterogeneity of 90% (Cochran's Q=9.89, P=0.002). The area of each square is proportional to the weight that the individual study contributed to the meta-analysis. Weights are from the random-effects analysis.

A) Allelic model

Study name	Odds ratio	Lower limit	Upper limit	Z-Value	p-Value
Monteleone 2008.	0,98	0,67	1,45	-0,09	0,929
Kolomeichuk 2014.	0,62	0,51	0,76	-4,71	0,000
Galbete 2012.	1,17	0,95	1,44	1,48	0,138
Li 2020.	3,42	2,10	5,55	4,96	0,000
	1,21	0,69	2,13	0,66	0,506

Statistics for each study — Odds ratio and 95% CI

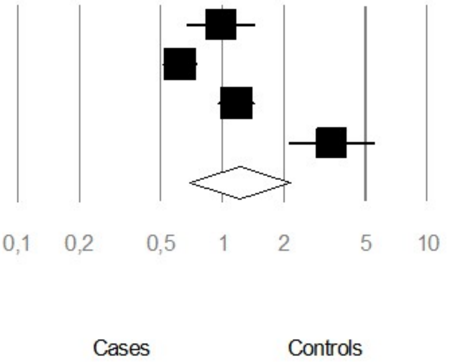

B) Dominant model

Study name	Odds ratio	Lower limit	Upper limit	Z-Value	p-Value
Monteleone 2008.	1,05	0,43	2,54	0,10	0,918
Galbete 2012.	1,28	0,80	2,06	1,02	0,306
Kolomeichuk 2014.	0,50	0,34	0,75	-3,34	0,001
Li 2020.	9,67	1,28	73,04	2,20	0,028
	1,11	0,51	2,41	0,26	0,797

Statistics for each study — Odds ratio and 95% CI

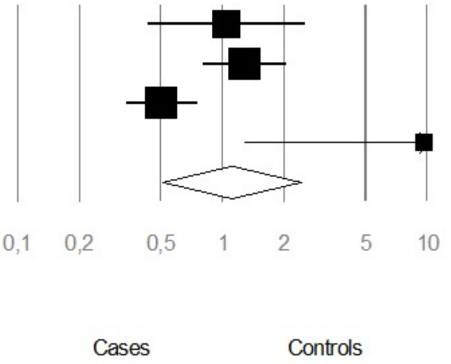

C) Recessive model

Study name	Odds ratio	Lower limit	Upper limit	Z-Value	p-Value
Monteleone 2008.	0,98	0,59	1,61	-0,09	0,929
Galbete 2012.	1,20	0,92	1,57	1,35	0,177
Kolomeichuk 2014.	0,56	0,43	0,74	-4,10	0,000
Li 2020.	3,87	2,25	6,67	4,87	0,000
	1,23	0,62	2,43	0,60	0,548

Statistics for each study — Odds ratio and 95% CI

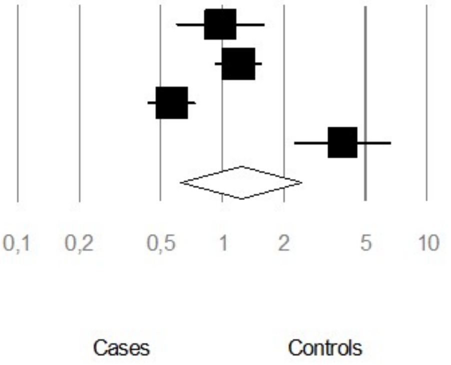

Figure S2. Forest plot for the association between metabolic syndrome and *CLOCK* gene rs1801260 polymorphism. Panel A) Forest plot for the association between metabolic syndrome and allelic model of rs1801260 polymorphisms with the heterogeneity of 85% (Cochran's Q=48.18, I^2=93.78%, P<0.001). B) Forest plot for the association between metabolic syndrome and dominant model of rs1801260 polymorphisms with the heterogeneity of 81% (Cochran's Q=15.03, I^2=80.05%, P=0.001). Panel C) Forest plot for the association between metabolic syndrome and recessive model of rs1801260 polymorphisms with the heterogeneity of 92% (Cochran's Q=42.35, I^2=92.92%, P<0.001). The area of each square is proportional to the weight that the individual study contributed to the meta-analysis. Weights are from the random-effects analysis.

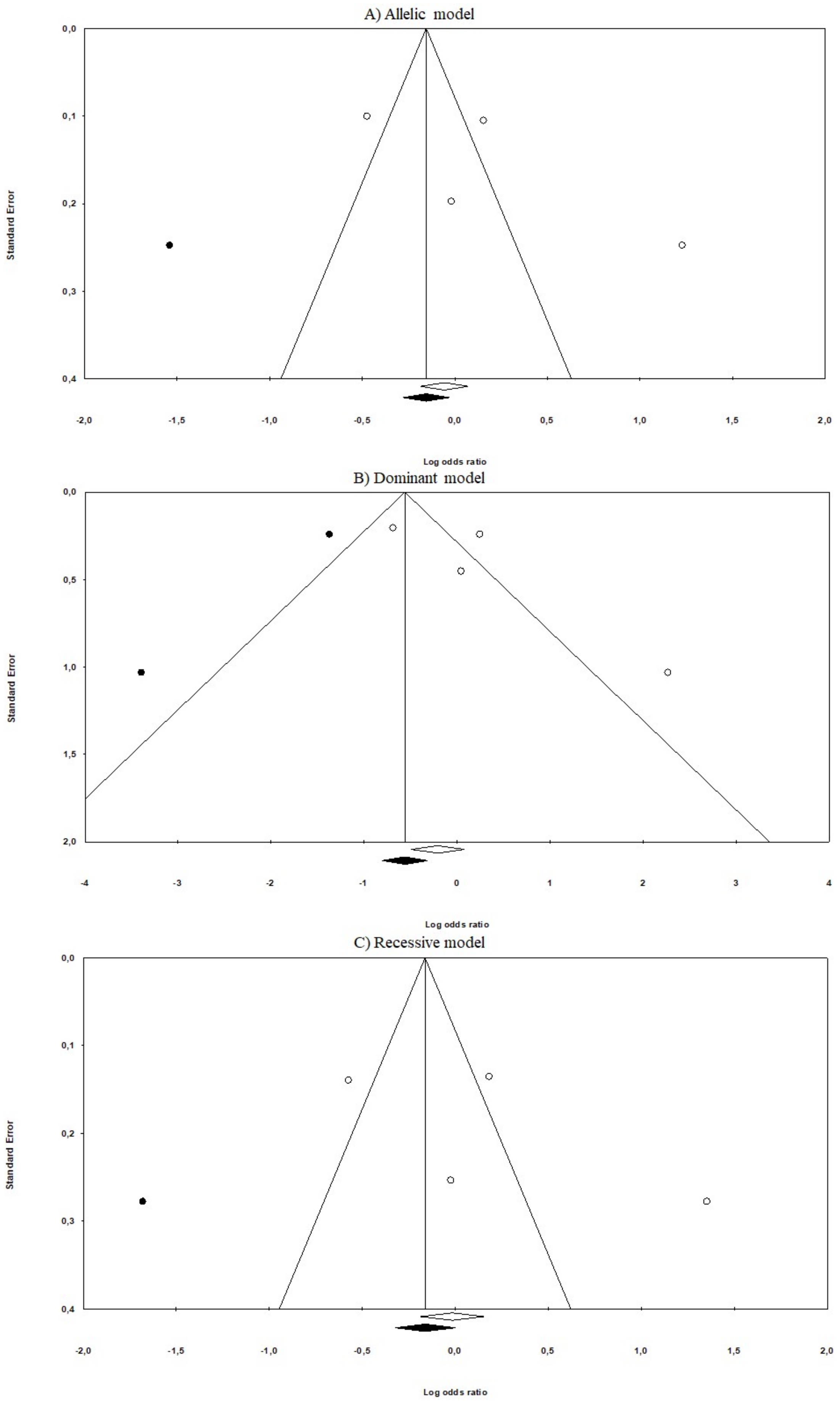

Figure S3. Funnel plot of meta-analysis of the *CLOCK* gene rs1801260 polymorphism. A) For the allelic model, B) for the dominant model, C) for the recessive model. Black circles denote imputed studies, trim-and-fill adjustment for publication bias.
